# Whole-body MRI for a patient with progressive multiple myeloma

**DOI:** 10.1093/bjrcr/uaaf028

**Published:** 2025-05-08

**Authors:** Jonathan E Henning, Taiga Nishihori, Ciara Freeman, Alexander Lazarides, Jinming Song, Davis Kuruvilla, Sebastian Feuerlein, James R Costello

**Affiliations:** University of South Florida Morsani College of Medicine, Tampa, FL 33602, United States; Department of Blood & Marrow Transplant and Cellular Immunotherapy, H. Lee Moffitt Cancer Center and Research Institute, Tampa, FL 33612, United States; Department of Blood & Marrow Transplant and Cellular Immunotherapy, H. Lee Moffitt Cancer Center and Research Institute, Tampa, FL 33612, United States; Department of Sarcoma, H. Lee Moffitt Cancer Center and Research Institute, Tampa, FL 33612, United States; Department of Hematopathology, H. Lee Moffitt Cancer Center and Research Institute, Tampa, FL 33612, United States; University of South Florida Morsani College of Medicine, Tampa, FL 33602, United States; Department of Diagnostic Imaging and Interventional Radiology, H. Lee Moffitt Cancer Center and Research Institute, Tampa, FL 33612, United States; Department of Diagnostic Imaging and Interventional Radiology, H. Lee Moffitt Cancer Center and Research Institute, Tampa, FL 33612, United States

**Keywords:** multiple myeloma, progressive, whole-body MRI

## Abstract

Multiple Myeloma represents a plasma cell disorder that can result in hallmark bony destructive change in addition to other signs of myelomatous disease. Imaging often helps in establishing the diagnosis and staging the patient. There are several different imaging modalities that can provide different levels of insight into the disease extent. We report a unique case of multiple myeloma where the progressive nature of the patient’s disease highlights the strengths and limitations of the different imaging approaches. Whole-body MRI represents a noncontrast imaging technique that directly images the bone marrow space, allowing for disease detection that can precede the onset of cortical and trabecular destructive changes. In so doing, whole-body MRI provides a level of insight that far exceeds traditional plain films and even CT. Using the findings from many different imaging modalities (plain films, CT, PET-CT, and whole-body magnetic resonance imaging), we will discuss how imaging can help clinicians to better assess the patient’s disease burden and complement the foundations of traditional disease monitoring (serology, histopathology from biopsy, direct clinical exam, and observation).

## Introduction

Multiple myeloma (MM) is part of a spectrum of disorders involving clonal plasma cell proliferation and an increase in monoclonal immunoglobulins. The stages of plasma cell proliferation that precede MM are monoclonal gammopathy of undetermined significance (MGUS) and smoldering myeloma (SMM). Monoclonal gammopathy of undetermined significance represents an asymptomatic stage with a low risk of malignancy, while SMM is an asymptomatic, intermediate stage between MGUS and MM.^1^ Proliferation of plasma cells can eventually manifest in end-organ damage with an association with hypercalcemia, renal dysfunction, anaemia, or lytic bone lesions (often referenced by the acronym CRAB).^2^

Imaging functions as an important tool to aid in a diagnosis of MM. There are multiple modalities that can help visualize myelomatous disease. A conventional skeletal survey (CSS) has traditionally been ordered for the diagnosis and follow up evaluation of MM patients, but numerous limitations have led researchers to pursue more advanced imaging options such as CT, PET-CT, or MRI. Whole-body, low-dose computed tomography (WBLDCT) allows for enhanced visualization of bone lesions with a level of sensitivity (SN) that far exceeds CSS. Additionally, WBLDCT is performed with radiation levels that mirror that of a CSS and as low as 1.5 mSv.^3^ Through the detection of metabolically active disease, PET-CT is another diagnostic modality to identify myelomatous lesions and to monitor therapeutic response. Though these tools remain useful, whole-body magnetic resonance imaging (WBMRI) holds promise as the most accurate modality for evaluating MM. Since it provides the most sensitive and highly specific method to detect early bone marrow involvement, WBMRI can help establish an earlier diagnosis of MM. Towards this end, WBMRI has received endorsements from numerous consensus groups, including the International Myeloma Working Group (IMWG).^3^ With a known and established diagnosis of MM, WBMRI delivers the most detailed visualization of the disease extent including both osseous and extra-osseous involvement.

## Case report

**Figure 1.**
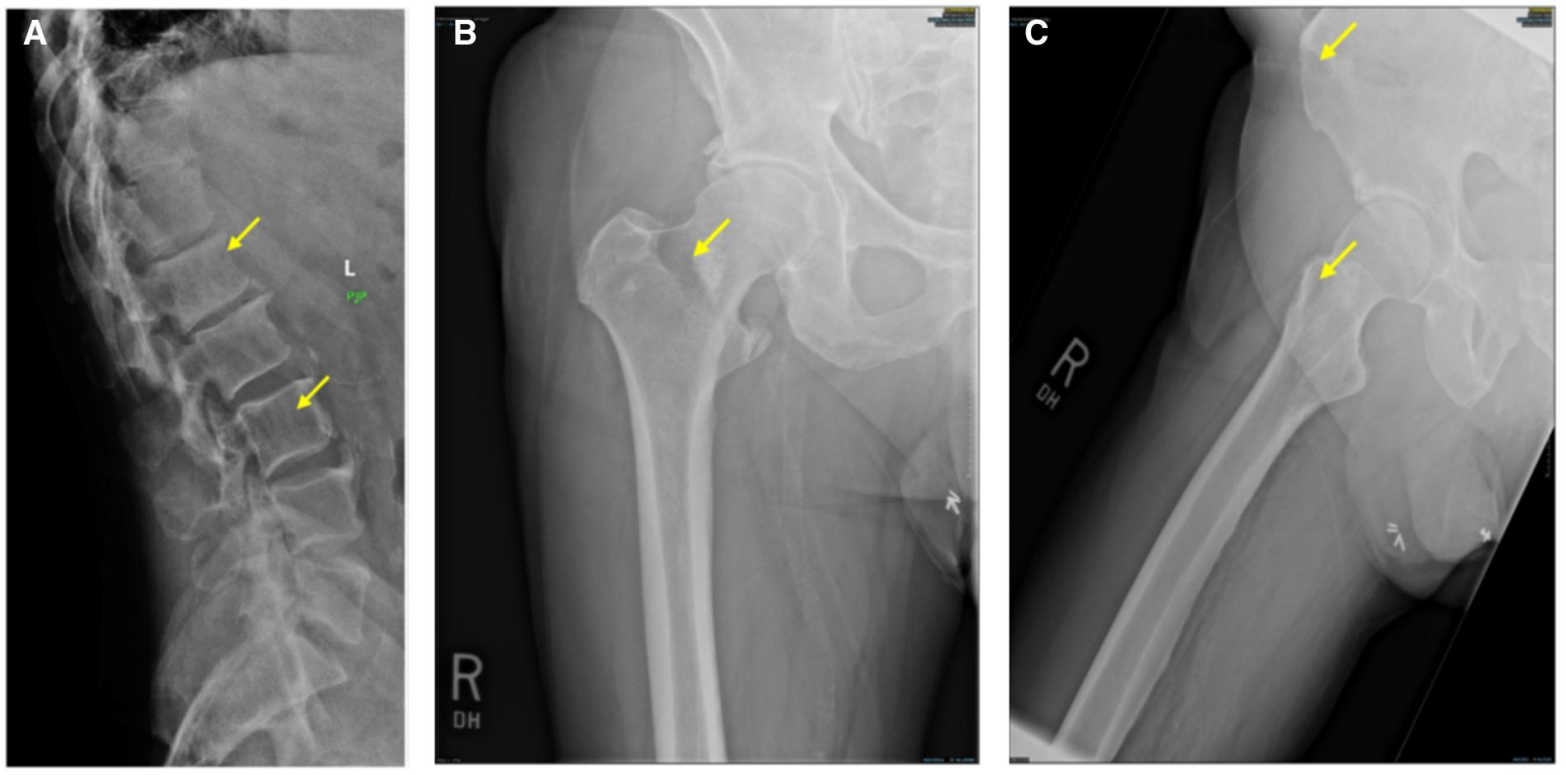
(A) Conventional Skeletal Survey. Destructive lytic lesions are present at the L1 and L3 vertebral bodies (arrows). (B and C) Destructive lytic lesions are visible in the femoral neck (B, arrow) and anterior iliac crest (C, arrow).

**Figure 2.**
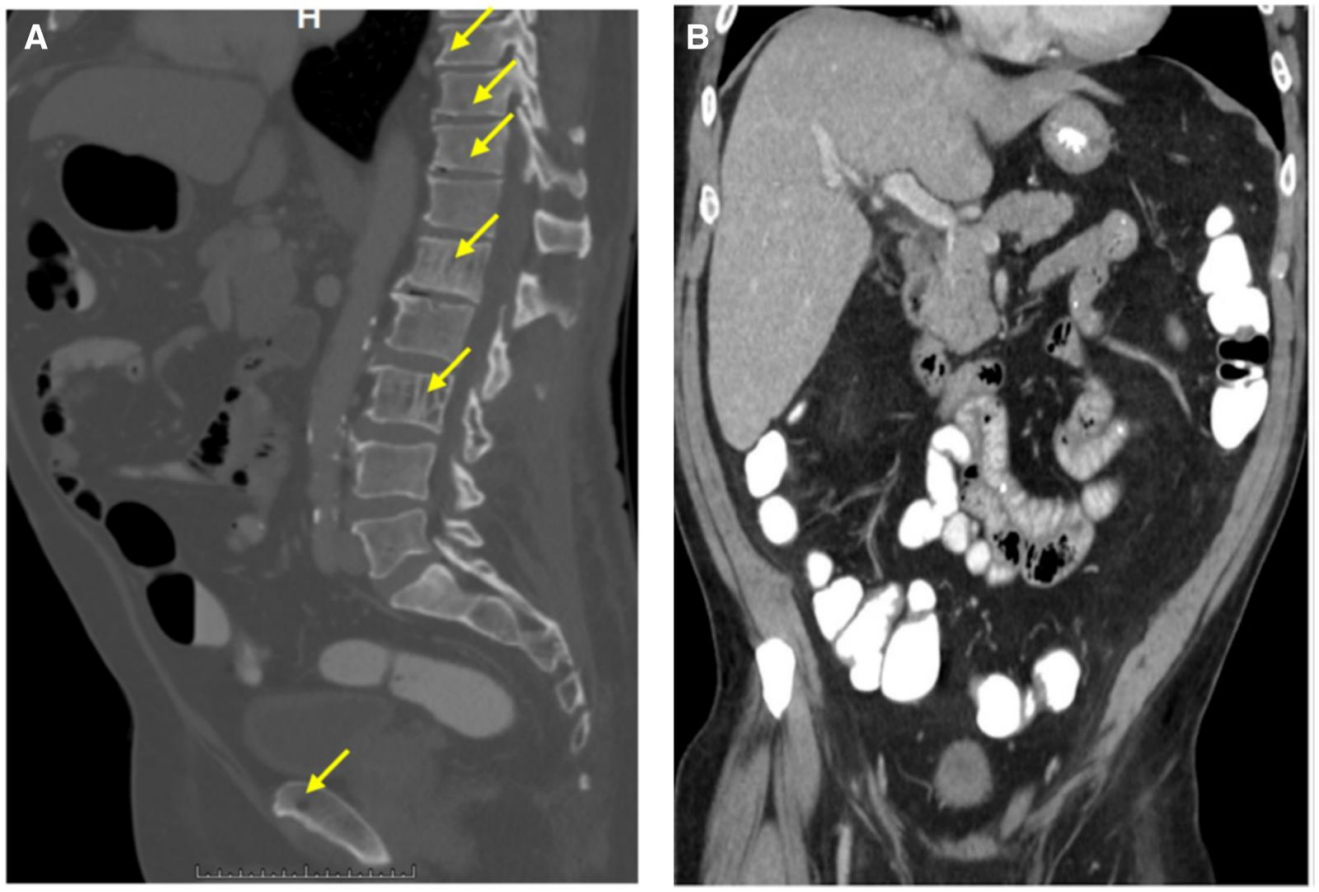
Whole-body, low-dose computed tomography (A) Vertebral lesions visualized on skeletal survey are also present on CT. In addition, CT demonstrated more osseous findings (arrows). (B) Unlike whole-body magnetic resonance imaging, the CT did not detect hepatic lesions.

A 73-year-old male with a diagnosis of MM had a long history of treatment at another institution. At the time of the patient’s initial diagnosis in 2005, his marrow demonstrated 24% plasma cells, an IgG level of 2400 mg/dL, and a skeletal survey that showed multiple lesions including pronounced lytic change at L1 and L3 (Figure 1A). These osseous changes were also noted on CT imaging (Figure 2A). In the coming years, the patient received many treatment regimens, including: (1) radiation therapy to the L3 level and an associated paraspinal mass (2) 4 cycles of bortezomib, doxorubicin, and dexamethasone (3) high-dose melphalan followed by autologous hematopoietic cell transplantation (4) thalidomide and dexamethasone (5) 4 cycles of bortezomib, lenalidomide, and dexamethasone. Over an extended timeframe, the patient showed varying levels of response to these treatments, but eventually, outside clinicians questioned the development of progressive disease. At this point, the IgG level was measured at 2633 mg/dL, and new lytic bone findings included a 4.2 cm right humeral head lesion with pronounced cortical erosion and destructive change. Subsequently, daratumumab, bortezomib, and dexamethasone treatment was administered, which showed an initial response with the IgG level dropping to 1281 mg/dL. Following the final dose, the IgG level then precipitously rose to approximately 5000 mg/dL.

**Figure 3.**
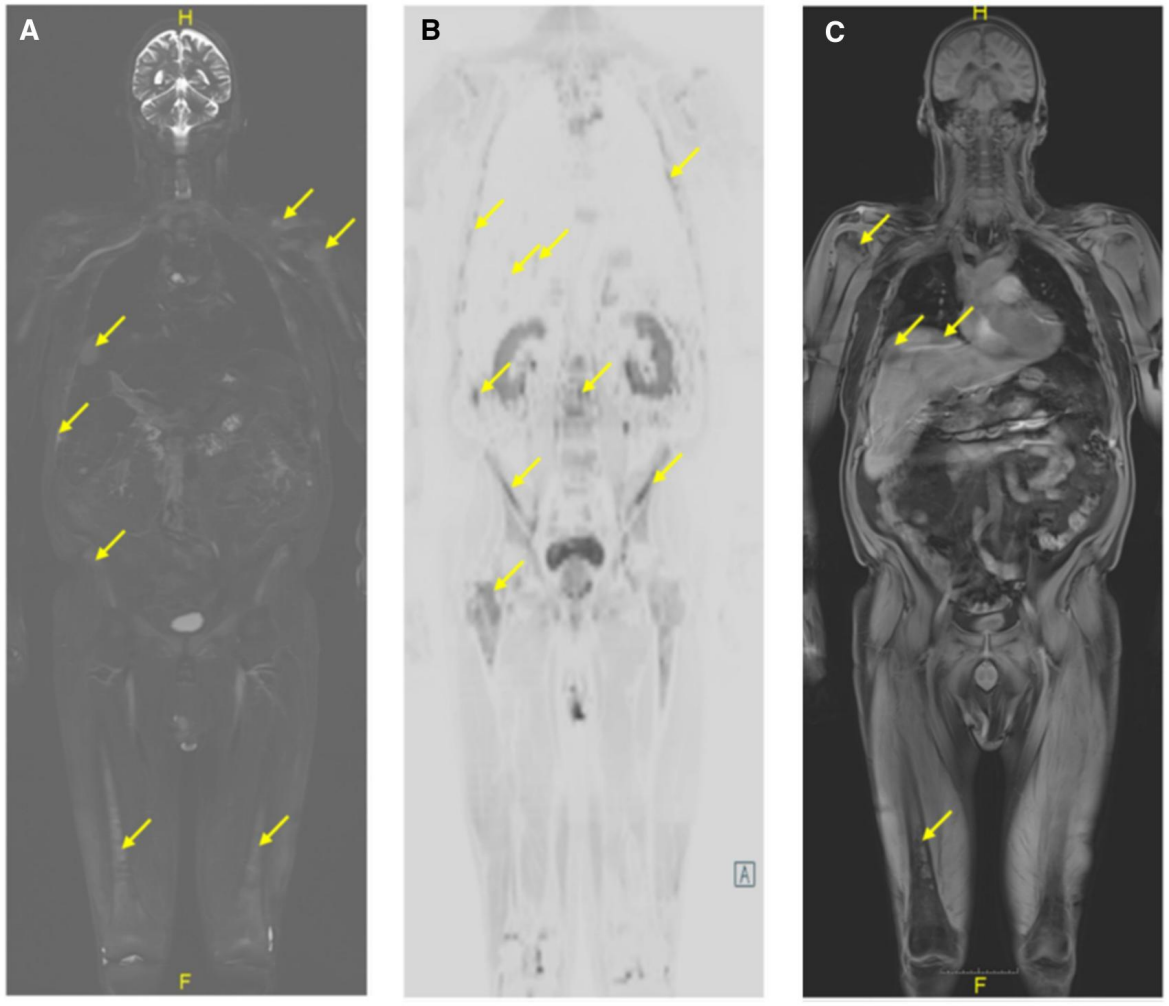
Whole-body magnetic resonance imaging (A) Coronal T2w short tau inversion recovery. Widespread disease is present, including both skeletal and hepatic lesions (arrows). (B) Inverted high *b*-value, coronal diffusion-weighted imaging highlighting diffuse disease (arrows). (C) Coronal Dixon (water only). Similar diffuse involvement of the liver and osseous structures is evident (arrows).

**Figure 4.**
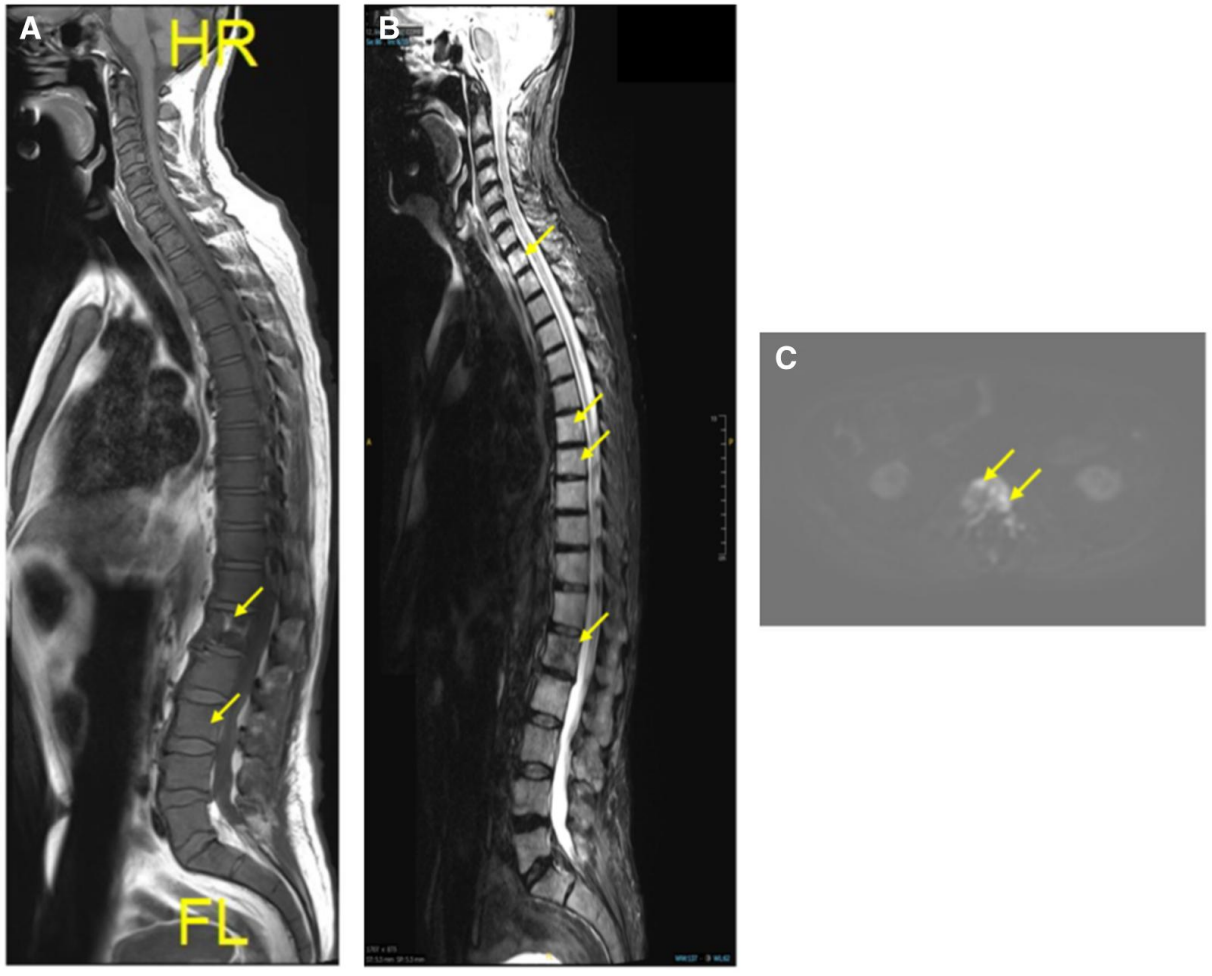
Whole-body magnetic resonance imaging (a) Sagittal T1w. There are several sites of disease within the lumbar spine (arrows). (b) Sagittal, T2w short tau inversion recovery highlighting T2 hyperintense disease in the spine (arrows). (c) High *b*-value diffusion-weighted imaging showing high signal at L3 (arrows).

**Figure 5.**
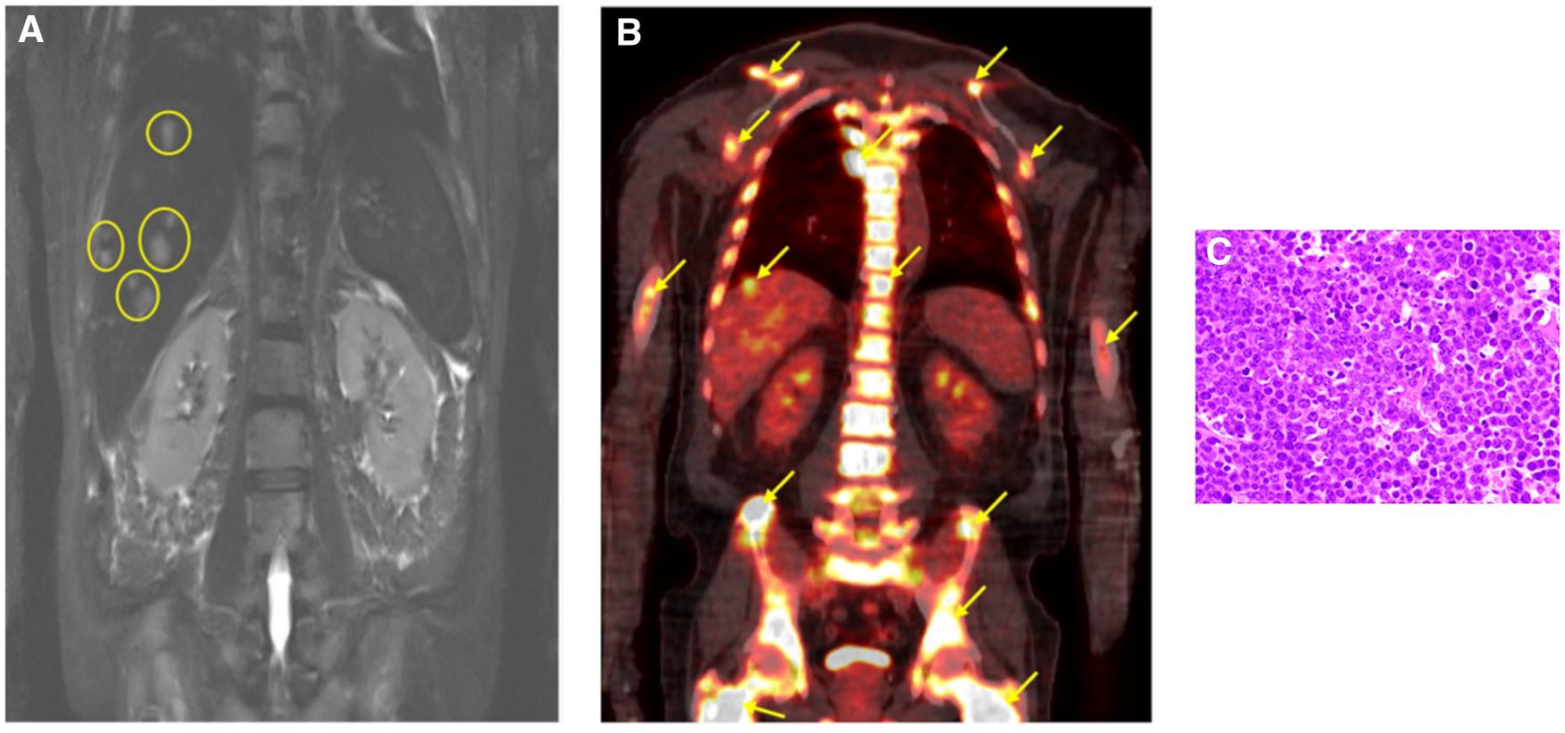
(A) Whole-body magnetic resonance imaging T2w short tau inversion recovery. Several more hepatic lesions (arrows) are present compared to PET-CT. (B) PET-CT. Widespread hypermetabolic activity including osseous and hepatic lesions (arrows). (C) Core biopsy (10×) showing markedly hypercellular bone marrow (∼100%) with diffuse replacement by plasma cell myeloma.

A month after progressive disease was first suspected, the patient presented to our institution with intense hip pain. A lytic lesion of the femoral neck and right anterior iliac crest was detected on radiograph (Figure 1B and C). Lab results following this visit showed elevated levels of monoclonal spike protein (M-spike) at 3.5 g/dL, IgG level at 4662 mg/dL, and a high level of kappa free light chains at 2675 mg/dL. Clinicians were even more concerned with progressive disease with increasing osseous involvement, and a whole-body MRI (protocol detailed in Table 1) and PET-CT were ordered. Whole-body MRI findings included extensive disease involvement throughout both the axial and appendicular skeleton, highlighted by T2 hyperintense foci on the short tau inversion recovery (STIR) imaging with associated diffusion restriction on the apparent diffusion coefficient (ADC) map, described by dark hypointense signal below 1000 µm^2^/second. On T2 STIR, the widespread myelomatous involvement included both skeletal and hepatic lesions. These skeletal lesions were identified within the bilateral humeral heads, femurs, scapula, and ribs (Figure 3A). Diffusion-weighted imaging and Dixon imaging showed similar patterns of diffuse disease (Figure 3B and C). Whole-body MRI findings were most pronounced at L1 and L3, where there was low T1 signal, high T2 STIR, and high signal on high *b*-value diffusion weighted imaging (DWI) with accompanying low signal on ADC map (Figure 4). When compared to the prior CT, there had been interval development of multiple new T2 hyperintense foci throughout the liver, consistent with diffuse extramedullary myelomatous involvement (Figure 5A). This extensive hepatic disease was notably occult on the CT (Figure 2B). Mirroring many of the findings seen on the WBMRI, diffuse osseous and extra-osseous involvement was also evident on the PET-CT. This included multiple hyper-metabolic findings within the liver in addition to extensive osseous involvement: (1) ribs with associated expansile and destructive soft tissue, (2) ilium, (3) sternum, (4) scapula, and (5) spine (Figure 5B). There were some sites of disease involvement seen on WBMRI that were even occult on PET-CT. This included a far more extensive description of disease involvement within the liver (Figure 5A and B).

**Table 1.** Our institutional WBMRI protocol^9^

Series	Coronal T1 dixon (vertex to knees) with generation of 2 PT fat fraction map	T2 coronal STIR (vertex to knees)	Axial T2w haste (vertex to knees)	Axial DWI (*b* values of 50 and 800 s/mm^2^; 1400 calculated) w/ADC maps (vertex to knees) with coronal reformats	T1w sagittal spine
Position	Supine	Supine	Supine	Supine	Supine
Image mode	3D	2D	2D	2D	2D
Thickness	5 mm	5 mm	5 mm	5 mm	4 mm
In-plane resolution	1.69 mm	0.949219 mm	1.25312 mm	1.72015 mm	0.625 mm
TR (ms)	4.24	1600	1200	7722	600
TE (ms)	1.34	85	179	60	9.5
Field strength	3T	3T	3T	3T	3T

This is adapted from the 2019 MY-RADS recommendations.

Abbreviation: ADC = apparent diffusion coefficient; DWI = diffusion weighted imaging; MY-RADS = myeloma response assessment and diagnosis system; STIR = short tau inversion recovery.

The patient showed extensive signs of osseous destructive change with myelomatous infiltration at many sites. The extent of extra-osseous disease and osseous involvement with cortical and trabecular destructive change supported a staging of progressive MM. This conclusion was further corroborated by the highly elevated IgG levels, rising serum free light chains levels, and a core biopsy revealing a markedly hypercellular bone marrow, completely replaced by plasma cell myeloma (Figure 5C).

## Discussion

We present a unique case of MM with diffuse disease that shows very significant osseous and extra-osseous involvement. The progressive nature of the patient’s disease helps to highlight the strengths and limitations of the different imaging modalities.

Several imaging options are available for patients who are suspected to have MM or already have an established diagnosis. Conventional skeletal survey is the traditional technique for visualizing lytic foci within bone. In addition to being time consuming (image acquisition time of at least 30 minutes) and poorly tolerated by patients, false negative (FN) results are common. In order to visualize a lytic finding by CSS, there must already be considerable signs of lytic bony destruction.^4^ For our patient, the CSS (Figure 1) detected far fewer lytic findings in comparison to the subsequent CT (Figure 2). Whole-body, low-dose computed tomography is faster, cost-effective, relatively sensitive for detection of lytic foci, and boasts a low radiation dose profile, nearly equivalent to CSS (1.5-2.4 mSv).^4^ Ultimately, CSS should only be used when all other imaging options are unavailable, and it remains a last resort imaging option.

Despite the convenience of WBLDCT, WBMRI is radiation free and provides far better soft tissue contrast to enhance visualization of early disease. By directly imaging the bone marrow space through a selection of different imaging sequences (Dixon water only imaging, fat fraction maps, T2 STIR, DWI, T2 half-Fourier acquisition single-shot turbo spin-echo, T1w turbo-spin-echo), WBMRI demonstrates the highest overall SN for the detection of focal myelomatous disease.^5^ By using many different methods to directly image the marrow space, MRI can detect much earlier signs of disease than appreciated by WBLDCT or CSS. In our patient’s case, the WBMRI highlighted sites of disease involvement within the liver that were completely occult on WBLDCT. Furthermore, the WBMRI was able to illustrate far greater osseous involvement within the bone marrow that had not yet resulted in destructive lytic change. Such sites are completely inapparent on CSS and frequently occult on WBLDCT. Overall, this example underscores the clinical utility of WBMRI.

PET-CT is another valuable tool for the assessment of MM and the detection of focal lesions. While a very powerful technique that highlights metabolically active disease, PET-CT can fail to illustrate some sites of disease better appreciated on WBMRI, leading to higher FN rates and decreasing the overall SN of the PET-CT. In slightly more than 10%-15% of patients, current published literature reports that sites of disease with low expression of the glycolysis enzyme, hexokinase-2, are associated with higher FN rates.^6^ Recent unpublished data and experience would suggest that this incidence may be even higher. In instances where patients exhibit negative PET-CT results despite suspected disease presence, the underlying potential of low hexokinase-2 expression should be considered, and WBMRI could be advocated for a more comprehensive assessment of the disease. For our patient, the PET-CT showed many similar findings to the WBMRI, but there were some sites within the skeleton and liver (Figure 5B) where the extent of disease was still underrepresented by PET-CT. Given the potential risk of a FN result, WBMRI has received specific endorsements from the IMWG, National Institute of Clinical Excellence, and the British Society for Haematology. For example, the IMWG recommends WBMRI for first-time assessment of asymptomatic patients, SMM, and solitary bone plasmacytomas.

When screening is performed with WBMRI, the earlier detection of focal myelomatous disease can be associated with an increase in quality-adjusted life years compared to screening with CSS, CT, or PET-CT.^3^ This provides a powerful endorsement for the use of WBMRI in patients with MGUS and SMM and explains why the IMWG has recommended WBMRI as a first-choice screening tool. Patients with an established diagnosis of MM also benefit from WBMRI as a method to assess treatment response. The functional imaging of WBMRI (DWI) coupled with its multisequence analysis provides powerful tools to complement serology and clinical assessment. Such functional imaging sequences as DWI and its mathematical generation of an ADC map have assumed even greater importance. Diffusion weighted imaging and ADC maps ushered in the first wave of MRI-based quantitative imaging biomarkers to quantitatively detect early changes in tissue biophysical parameters and to enhance the evaluation of micro-environmental changes which were otherwise undetectable by such traditional imaging modalities as CSS and CT. The imaging and diagnostic benefits of WBMRI also extend to other disease processes. Numerous consensus societies advocate its use for imaging of leukemia/lymphoma, prostate cancer, melanoma, testicular cancer, ovarian cancer, myxoid liposarcoma, neurofibromatosis, and Langerhans cell histiocytosis, as well as screening for hereditary cancer predisposition syndromes (Li Fraumeni syndrome, hereditary pheochromocytoma/paraganglioma, constitutional mismatch repair deficiency).^7^

Despite this advocacy, the limitations of WBMRI should still be considered in this discussion. Its expensive cost, lower accessibility, and time consumption can present challenges. Currently, PET-CT is endorsed as the preferred imaging modality to assess treatment response, but these recommendations are based on early work that focused on comparing the SN of DWI to PET-CT as opposed to accounting for the inherent composite value of a complete WBMRI exam, replete with multisequence and functional imaging in addition to high-contrast fat-fraction maps. Recent published literature has emphasized the importance of performing fat fraction maps with its added insight to distinguish early signs of disease from a background of red marrow conversion, to differentiate diffusely diseased bone marrow from focal disease on a background of diffuse involvement, to identify early treatment response, and to detect the very first imaging signs of disease recurrence. With the introduction of novel treatments such as chimeric antigen receptor T-cell therapy (CAR-T), the importance of adding such high contrast imaging sequences has become even more impactful.^8^ Empowered with functional imaging biomarkers that detect small variations in soft tissue composition and background biophysical quantitative signatures, WB-MRI signifies a “game changing” tool to perform more accurate and reproducible tumor response assessment.

## Learning points

In the assessment of MM, imaging is a helpful tool to further empower serology, biopsy, direct clinical exam, and clinical acumen.

- Conventional skeletal survey is cumbersome for patients and time-consuming. Coupled with its reduced SN, CSS should only be used when other options are unavailable.
- Whole-body, low-dose computed tomography is a useful first-line imaging approach for detecting bone disease, offering a well-tolerated and relatively sensitive approach for viewing lesions.
- Though WBLDCT is cost-effective and convenient, WBMRI offers the best visualization of the bone marrow space.
- Because of the enhanced SN and specificity for the detection of focal lesions, screening with WBMRI can allow for earlier diagnosis of MM. This is particularly powerful in establishing the diagnosis of MM for patients with MGUS and SMM, in addition to monitoring for treatment response for patients receiving novel therapeutics such as CAR-T.
- PET-CT is another useful approach for imaging evaluation, focal disease detection, and treatment response. Attention should be heeded to its limitations in identifying diseases characterized with low expression of hexokinase-2.
